# Iodine and other factors associated with fertility outcome following oil-soluble contrast medium hysterosalpingography: a prospective cohort study

**DOI:** 10.3389/fendo.2024.1257888

**Published:** 2024-06-20

**Authors:** Divya M. Mathews, Jane M. Peart, Robert G. Sim, Neil P. Johnson, Susannah O’Sullivan, José G B Derraik, Paul L. Hofman

**Affiliations:** ^1^ Liggins Institute, University of Auckland, Auckland, New Zealand; ^2^ Starship Children’s Hospital, Health New Zealand | Te Whatu Ora, Auckland, New Zealand; ^3^ Auckland Radiology Group, Auckland, New Zealand; ^4^ Robinson Research Institute, University of Adelaide, Adelaide, SA, Australia; ^5^ Department of Obstetrics and Gynaecology, Faculty of Medical and Health Sciences, University of Auckland, Auckland, New Zealand; ^6^ Repromed Auckland, Auckland, New Zealand; ^7^ Endocrinology, Greenlane Clinical Centre, Auckland District Health Board, Auckland, New Zealand; ^8^ Department of Paediatrics: Child & Youth Health, Faculty of Medicine and Health Sciences, University of Auckland, Auckland, New Zealand; ^9^ Environmental–Occupational Health Sciences and Non-Communicable Diseases Research Group, Research Institute for Health Sciences, Chiang Mai University, Chiang Mai, Thailand; ^10^ Department of Women’s and Children’s Health, Uppsala University, Uppsala, Sweden

**Keywords:** fertility, hysterosalpingography, iodine, oil-soluble contrast medium, pregnancy, age, tubal patency, hypothyroidism

## Abstract

**Objective:**

To examine factors associated with fertility following hysterosalpingography (HSG) using an oil-soluble contrast medium (OSCM).

**Design:**

In a prospective cohort study on 196 women undergoing OSCM HSG, we showed that iodine excess was almost universal (98%) and mild subclinical hypothyroidism was frequent (38%). Here, we report the analyses of secondary outcomes examining factors associated with the likelihood of pregnancy following the HSG.

**Setting:**

Auckland, New Zealand (2019–2021).

**Sample:**

196 women with primary or secondary infertility who underwent OSCM HSG.

**Methods:**

Baseline and serial urine iodine concentrations (UIC) and thyroid function tests were measured over six months following the HSG. Pregnancy and treatment with levothyroxine during the study period were documented.

**Results:**

Following OSCM HSG, pregnancy rates were 49% in women aged <40 years (77/158) but considerably lower (16%) among those ≥40 years (6/38). Similarly, live birth rates were markedly lower in women ≥40 years (17%; 1/6) versus <40 years (73%; 56/77). 29% of participants were iodine deficient at baseline despite advice recommending iodine fortification. Following HSG, the likelihood of pregnancy in women with moderate iodine deficiency was 64% higher than in women with normal iodine levels (p=0.048). Among women aged <40 years who had subclinical hypothyroidism (n=75), levothyroxine treatment was associated with higher pregnancy rates compared to untreated women [63% (26/48) vs 37% (10/27), respectively; p=0.047].

**Conclusion:**

OSCM HSG was associated with higher pregnancy rates in women ≤40 than in those aged >40 years. Iodine deficiency was relatively common in this cohort, and increased iodine levels from OSCM exposure may contribute to the improved fertility observed with this procedure.

**Trial registration:**

This study is registered with the Australian New Zealand Clinical Trials Registry (ANZCTR: 12620000738921) https://anzctr.org.au/Trial/Registration/TrialReview.aspx?ACTRN=12620000738921.

## Introduction

Hysterosalpingography (HSG) with oil-soluble contrast medium (OSCM) is known to improve pregnancy rates in women with infertility (1–3). Pregnancy rates of 39.7% and live birth rates of 38.8% were reported in the H2Oil study, the large multicentre trial that confirmed the fertility enhancement with OSCM HSG (3). Although the improvement in pregnancy rates was reported within the initial six months of the procedure (3–5), little is known about the characteristics of those women who achieved the greatest fertility benefit. A secondary outcome analysis of the H2Oil study could not identify any characteristics of women who would benefit from OSCM HSG (6). The paucity of data in this area partially reflects our lack of understanding of the mechanism(s) underlying the improved fertility observed with OSCM HSG.

Nonetheless, several mechanisms have been proposed, including a mechanical flushing effect (7), an immune-biological peritoneal bathing effect (8), and an immune-biological uterine bathing effect (9). The other hypothesis is that iodine in OSCM could contribute to this fertility improvement (10). The reasons behind this postulation are the association between iodine deficiency and infertility and the iodine excess state produced by OSCM exposure. OSCM, such as Lipiodol, contains approximately 480 mg/ml of iodine (11) and has a reported half-life of approximately 50 days (12), creating severe and prolonged iodine excess for six months post-procedure. Recently published research from our group suggested almost universal (98%) iodine excess following an OSCM HSG, leading to the frequent occurrence of subclinical hypothyroidism (38%; 71/188) and an occasional occurrence of late-onset hyperthyroidism (5%; 9/196) (**Supplementary Table 1**) (13).

While iodine uptake via sodium-iodide symporters occurs mainly in the thyroid, other tissues also actively take up iodine from circulation. Two such examples are ovaries and endometrium, which have relatively high levels of sodium-iodide symporters (14, 15). The effect of iodine on the function of the ovaries and endometrium remains unclear. Still, it seems likely to have an important role, as iodine deficiency and insufficiency are well-established causes of subfertility (16). This study aimed to examine factors associated with increased fertility and live births following OSCM HSG, particularly the potential effects of iodine status on pregnancy rates before and after the HSG.

## Methods

The SELFI (Safety and Efficacy of Lipiodol in Fertility Investigations) Study was a prospective cohort study conducted in the Auckland region, New Zealand (2019–2021) (17). 196 consecutively consenting women who underwent OSCM HSG were followed for 6 months. The study’s primary outcome was the development of subclinical hypothyroidism, and our findings on iodine excess and thyroid dysfunction following OSCM HSG have been published (13). Secondary outcomes related to fertility are discussed in this article.

The inclusion and exclusion criteria are listed in **Supplementary Table 2**. Details of the HSG protocol and investigations are available in the published protocol (17). Clinical parameters assessed at baseline (before the HSG) included urine iodine concentration (UIC), and serum concentrations of thyroid stimulating hormone (TSH), free thyroxine (FT4), free triiodothyronine (FT3), and anti-mullerian hormone (AMH). The OSCM used in the HSG procedure was Lipiodol Ultrafluide (Guerbet, Aulnay-Sous-Bois, France). Following the HSG, participants had UIC measured at weeks 1, 4, 12, and 24, and thyroid function tests (TSH, Free T4 and Free T3) done at weeks 1, 4, 8, 12, 16, 20, and 24 (**Supplementary Figure 1**). Biochemical pregnancy was defined as a positive beta human chorionic gonadotropin (β-hCG) test. Live births were recorded, and any thyroxine treatment initiated by their primary clinician during the study period was documented.

The associations between clinical parameters and the likelihood of biochemical pregnancy were assessed with generalised linear models using a modified Poisson procedure with robust error variances (18). Model outcomes were reported as the unadjusted relative risk (RR) or the adjusted relative risk (aRR) and their respective 95% confidence intervals (CI). Models were adjusted for TSH levels and UIC at baseline, woman’s age (<35 years/35–39.9 years/≥40 years), and the instilled OSCM volume. UIC AUC calculations and data analyses were performed using SAS v9.4 (SAS Institute, Cary, NC, USA). Figures were created in GraphPad Prism v8.2.1 (GraphPad Software Inc., San Diego, CA, USA). All statistical tests were two-tailed, with statistical significance maintained at the 5% level, with no adjustments for multiple comparisons (19). There was no imputation of missing values.

## Results

### Study population

**Table 1** describes the demographic characteristics of the study population at baseline (n=196). Participants had a median age of 36.2 years (range 26 to 49 years), with 38 (19%) women aged ≥40 years.

**Table 1:** Demographic and clinical characteristics of the SELFI Study participants at baseline prior to hysterosalpingography.

Characteristic	Parameter	Level	
**n**			196
**Demography**	**Age (years)**		36.2 [32.8, 39.3]
	**Ethnicity**	**NZ European/European**	118 (60.2%)
		**Indian**	37 (18.9%)
		**Other Asian**	32 (16.3%)
		**Māori**	5 (2.6%)
		**Pacific**	4 (2.0%)
**Clinical**	**BMI (kg/m^2^) ^1^**		23.9 [21.9, 27.2]
	**BMI status ^1,2^**	**Normal weight**	95 (62.5%)
		**Overweight**	12 (7.9%)
		**Obesity**	45 (29.6%)
	**TSH (mIU/L)**		1.8 [1.3, 2.5]
	**Urine iodine (μg/L) ^3^**		152 [89, 228]
	**Infertility cause**	**Idiopathic**	130 (66.3%)
		**Endometriosis**	37 (18.9%)
		**PCOS**	15 (7.7%)
		**Other**	14 (7.1%)
	**Infertility type ^4^**	**Primary**	147 (75.0%)
		**Secondary**	49 (25.0%)
	**Fertility treatment ^5^**	**None**	141 (71.9%)
		**IVF**	31 (15.8%)
		**IUI**	19 (9.7%)
		**Unknown**	5 (2.6%)
	**Iodine status ^3,6^**	**Deficiency**	52 (29.2%)
		** Severe**	nil
		** Moderate**	12 (23.1%)
		** Mild**	40 (76.9%)
		**Normal**	98 (55.1%)
		**Excessive**	28 (15.7%)

Data are n (%) or median [Q1, Q3]. BMI, body mass index; IUI, intra-uterine insemination; IVF, *in vitro* fertilisation; OSCM, oil-soluble contrast medium; PCOS, polycystic ovarian syndrome; TSH, thyroid-stimulating hormone.

^1^n=152.

^2^Normal weight was defined as a BMI ≥18.5 to <25 kg/m^2^; overweight as ≥25 to <30 kg/m^2^; and obesity as ≥30 kg/m^2^.

^3^n=183.

^4^Primary and secondary infertility were defined as at least 12 months of unsuccessfully attempting pregnancy with no previous live births and with a previous live birth, respectively (20).

^5^Treatment (if any) was undertaken after OSCM HSG.

^6^Iodine status was defined as per WHO criteria (21) using urine iodine concentrations: deficiency (<100 μg/L), severe deficiency (<20 μg/L), moderate deficiency (≥20 to <50 μg/L), mild deficiency (≥50 to <100 μg/L), normal (≥100 to <300 μg/L), and excessive (≥300 μg/L).

Based on WHO definitions of iodine status (21), 55% of participants were iodine sufficient, 29% were deficient, and 16% had iodine excess (**Table 1**). Among those who were iodine deficient, most (77%) had mild deficiency, and the rest (23%) had moderate deficiency (**Table 1**).

### Pregnancy rates following OSCM HSG

Overall, 83 participants (42%) had a biochemical pregnancy (i.e., a positive serum β-hCG result), while 57 (29%) had an ongoing pregnancy that progressed to a live birth. The other 26 participants (13%) had a miscarriage, usually in the first trimester. When only women aged 40 years or below were considered, 49% (77/158) conceived and 73% of them had a live birth (56/77), which equated to 37% of women in this age group (56/158).

**Figure 1:**
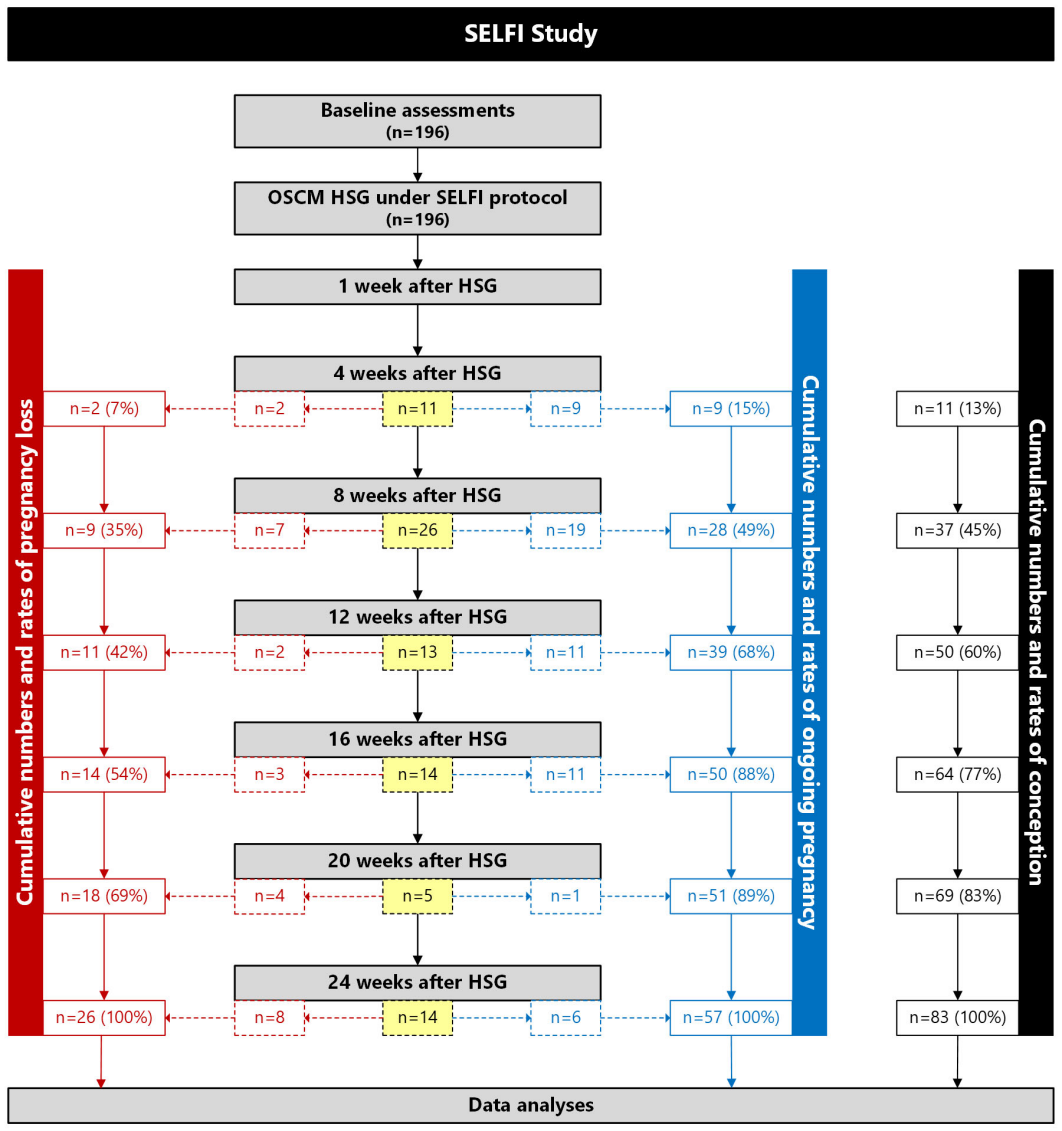
Flow diagram showing the numbers and cumulative rates of conception (black), pregnancy loss (red), and ongoing pregnancies (blue) among women who underwent hysterosalpingography (HSG) with an oil-soluble contrast medium (OSCM) in the SELFI Study. Values within boxes with dashed lines are the numbers and rates of new conceptions, pregnancy losses, and ongoing pregnancies at a given time point since HSG. Values within boxes with solid lines are the cumulative numbers and rates of conceptions, pregnancy losses, and ongoing pregnancies at a given time point since HSG. The number in bold font (n=196) indicates the number of participants who underwent OSCM HSG and completed study (i.e., there were no participants lost to follow-up).

The timing of conception and subsequent miscarriages in association with the OSCM HSG procedure are itemised in **Figure 1**. Nearly half of all conceptions (45%; 37/83) were recorded within 8 weeks of the HSG, and more than three quarters (77%; 64/83) had occurred by week 16 (**Figure 1**). Notably, the vast majority of ongoing pregnancies (88%; 50/57) were recorded by week 16 (**Figure 1**).

An exploratory analysis showed no association between infertility cause and biochemical pregnancy rates following OSCM HSG (**Supplementary Table 3**). In addition, baseline iodine status did not differ between women with different infertility causes (**Supplementary Table 4**), and there was no evidence that iodine status differentially affected pregnancy rates in these groups (**Supplementary Table 5**). Similarly, there was no evidence that BMI (**Supplementary Table 6**) or assisted reproductive technologies (i.e., intrauterine insemination or *in vitro* fertilisation) (**Supplementary Table 7**) affected pregnancy rates.

### Woman’s age at baseline

**Figure 2:**
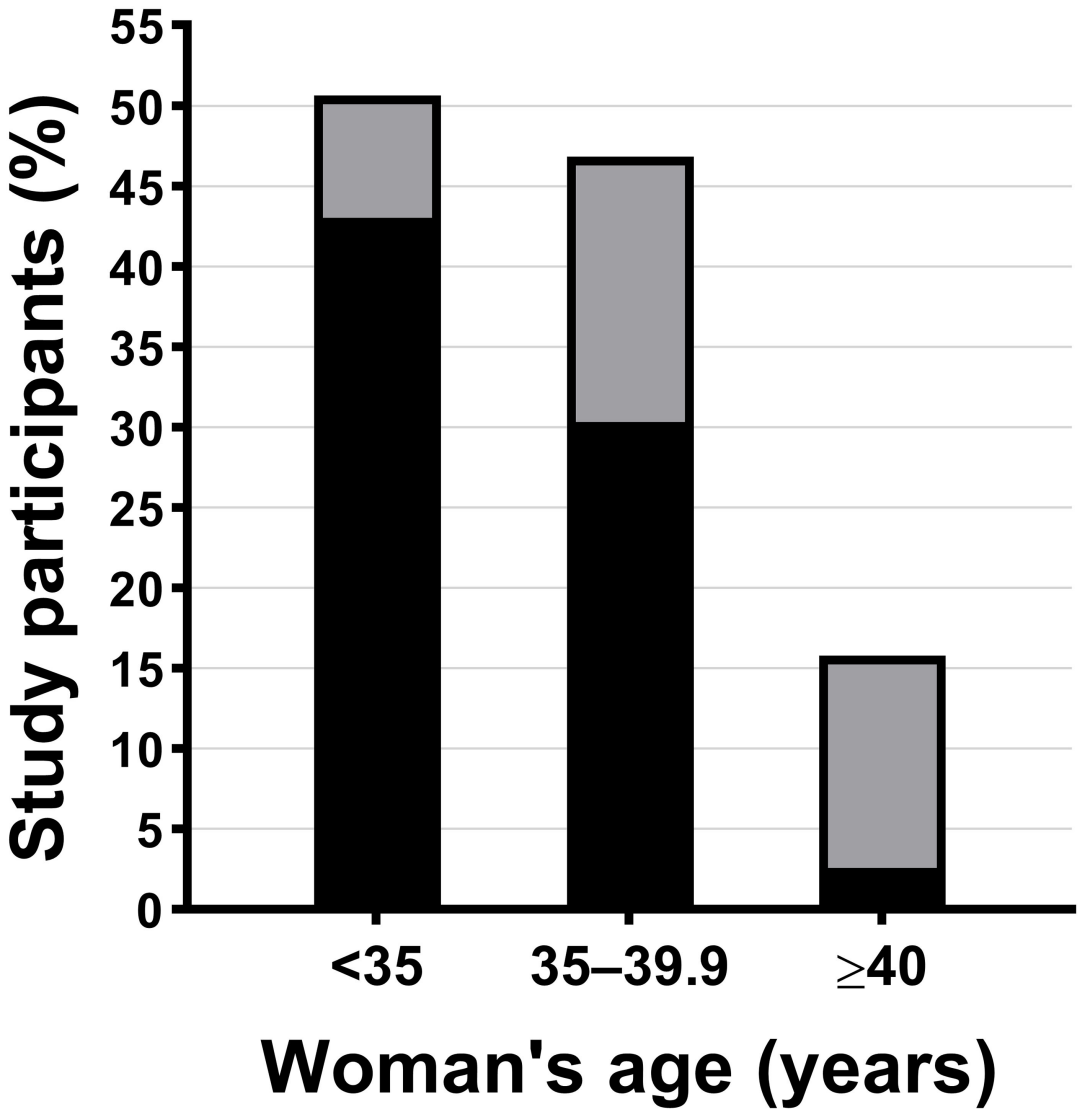
Biochemical pregnancy (based on beta human chorionic gonadotropin positivity) according to the woman’s age at baseline: <35 years (n=40), 35–39.9 years (n=37), and ≥40 years (n=6). The bands in each bar represent the percentage of women who conceived and either had a miscarriage (grey) or delivered a live baby (black).

Pregnancy rates were similar among women aged <35 and 35–39.9 years, but there was a marked decline in fertility rates among participants aged ≥40 (**Figure 2**; **Supplementary Table 8**). Only 16% (6/38) of the latter became pregnant compared to 51% (40/79) and 47% (37/79) of women aged <35 years and 35–39.9 years, respectively (p<0.001) (**Figure 2**; **Supplementary Table 8**). Thus, in comparison to the women aged ≥40 years, those aged <35 years were 3 times more likely to become pregnant [aRR=3.03 (95% CI 1.43, 6.45); p=0.004] and women aged 35–39.9 years 2.9 times more likely [aRR=2.92 (95% CI 1.37, 6.25); p=0.009]. The rate of miscarriage was 30% (25/83), and this rate progressively increased with the woman’s age, so that 83% of those aged ≥40 years (5/6) experienced pregnancy loss (**Figure 2**; **Supplementary Table 8**).

### Iodine status

Overall, lower iodine levels at baseline were associated with a greater likelihood of pregnancy. Women who became pregnant had baseline UIC 21% lower than those who did not become pregnant (95% CI -38%, -1%; p=0.042) (**Supplementary Table 9**), with an adjusted mean difference slightly greater [-23% (95% CI -40%, -2%); p=0.033]. As a result, a 10-fold lower UIC at baseline was associated with a 77% increase in the likelihood of pregnancy [aRR 1.77 (95% CI 1.11, 2.81); p=0.017].

**Figure 3:**
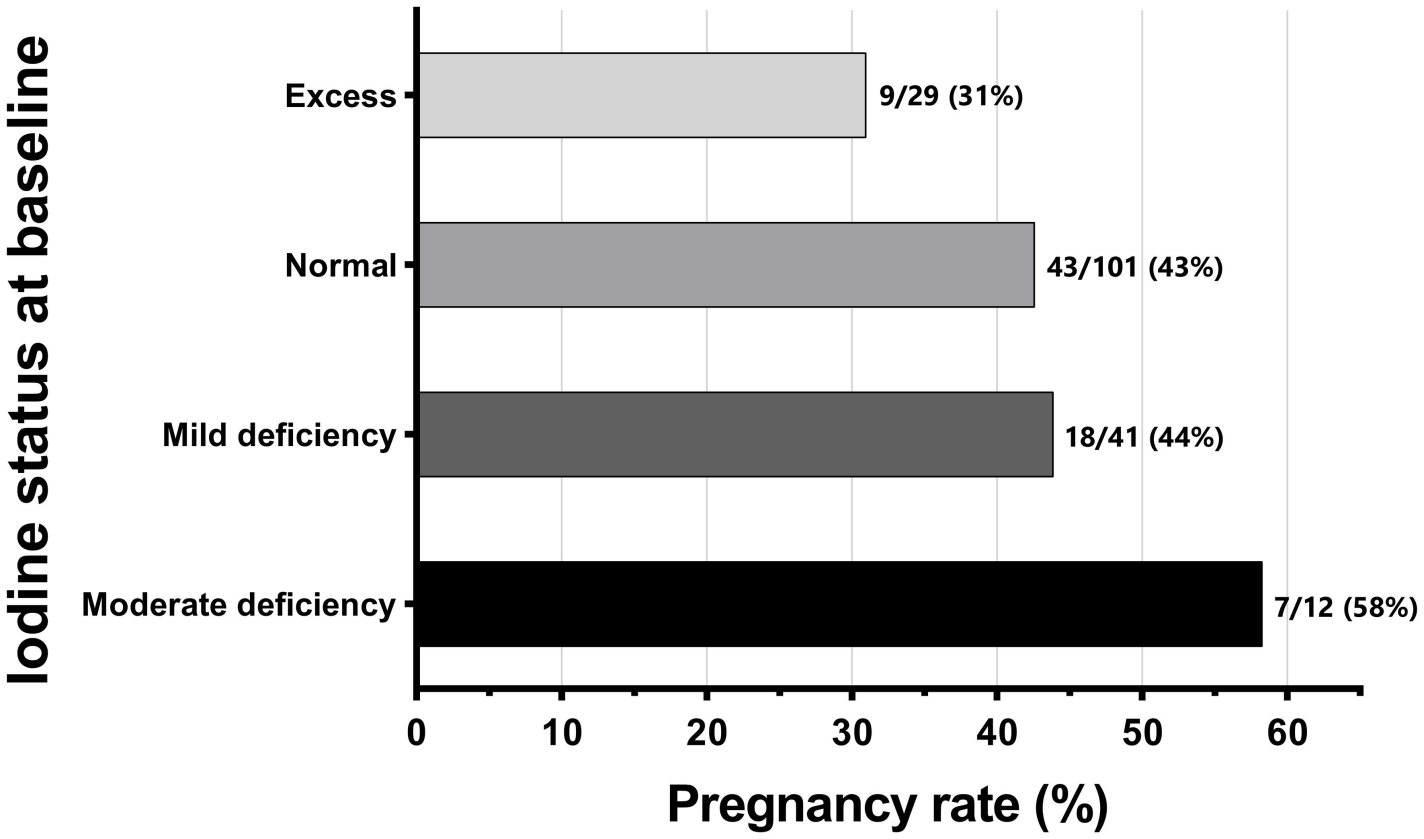
Frequencies and rates of biochemical pregnancy [based on beta human chorionic gonadotropin (β-hCG) positivity] according to the women's urine iodine status at baseline (n=183). Iodine status was classified according to WHO criteria: moderate deficiency (≥20 to <50 μg/L), mild deficiency (≥50 to <100 μg/L), normal (≥100 to <300 μg/L), and excess (≥300 μg/L) (21). No woman in the study had severe iodine deficiency (<20 μg/L).

Reflecting the above-described associations, pregnancy rates progressively decreased from the group of women with moderate iodine deficiency at baseline (58%) to those with excess iodine (31%) (**Figure 3**). Thus, the likelihood of pregnancy in women with moderate deficiency at baseline was 64% higher than in women with normal iodine levels [aRR 1.64 (95% CI 1.01, 2.67); p=0.048] and more than 2-fold higher than those with iodine excess [aRR 2.13 (95% CI 1.09, 4.14); p=0.026]. These data indicate that women with iodine deficiency treated by iodine exposure from OSCM HSG had improved pregnancy rates compared to those who were iodine-sufficient or had excess iodine. Interestingly, we also noted a higher pregnancy rate (43%) in the iodine-sufficient group compared to women with iodine excess (**Figure 3**).

### Iodine levels after the HSG

Iodine excess (UIC ≥300 μg/L) after the OSCM HSG was almost universal among our participants (98%) and was often marked (90% had UIC ≥1000 μg/L and 17% had UIC >10,000 μg/L) and prolonged (67% had UIC ≥1000 μg/L lasting at least three months) (13). However, in contrast to baseline iodine status, UIC after HSG did not seem to influence the likelihood of conception, with the UIC time-weighted area under the curve similar in women who did and did not conceive [45.4 mg/L/week (95% CI 35.2, 58.4) vs 42.5 mg/L/week (95% CI 34.9, 51.8); p=0.69].

### AMH levels at baseline

AMH concentrations (reflecting ovarian reserve) were correlated with the women’s age (*r*=-0.39; p<0.0001). Thus, AMH steadily declined with increasing age (**Supplementary Figure 2**). Women who became pregnant during the study had higher AMH levels compared to those who did not (22.4 vs 17 μg/L, respectively; p=0.021) and were nearly 2 years younger on average (p=0.003). There was no observed effect of the HSG on AMH levels.

### Treatment of subclinical hypothyroidism

Mild subclinical hypothyroidism (TSH 4-10 mIU/L with normal FT4) was the most common thyroid dysfunction in the SELFI cohort. The treatment of mild subclinical hypothyroidism with thyroxine is controversial (22), and during the SELFI Study, an individualised treatment decision was made by the participant’s primary clinician. There was a trend suggesting that women treated with levothyroxine were more likely to conceive compared to untreated women [54% vs 35%, respectively (**Table 2**); aRR 1.66 (95% CI 0.97, 2.84); p=0.063]. Notably, when only women aged <40 years at baseline were considered, the pregnancy rate after levothyroxine treatment was higher than that of untreated women (63% vs 37%; p=0.047; **Table 2**), with a 75% increase in the likelihood of pregnancy [aRR 1.75 (95% CI 1.01, 3.02); p=0.046].

**Table 2:** Rates of biochemical pregnancy based on beta human chorionic gonadotropin (β-hCG) positivity according to the diagnosis of subclinical hypothyroidism (SCH), the timing of its onset, and any subsequent thyroxine treatment.

	Subclinical hypothyroidism			Thyroxine		Thyroxine (aged <40 years)	
	No SCH	SCH at baseline	SCH after HSG	Not treated	Treated	Not treated	Treated
***n***	117 (59.7%)^1^	8 (4.1%)^1^	71 (36.2%)^1^	31 (39.2%)^2^	48 (60.8%)^2^	27 (41.5%)^2^	38 (58.5%)^2^
**β-hCG negative**	71 (60.7%)	2 (25.0%)	40 (56.3%)	20 (64.5%)	22 (45.8%)	17 (63.0%)	14 (36.8%)
**β-hCG positive**	46 (39.3%)	6 (75.0%)	31 (43.7%)	11 (35.5%)	26 (54.2%)	10 (37.0%)	24 (63.2%) *

HSG, hysterosalpingography.

Unless otherwise stated, data are *n* and percentage within a given column.

^1^Percentages from all study participants.

^2^Percentages from participants who had SCH at some point during the study.*p=0.047 from a Fisher's exact test.

### Tubal patency

The data on tubal patency status for our study participants are provided in **Supplementary Table 10**. Notably, 3 out of 16 women (19%) with radiological evidence of bilateral tubal obstructions had spontaneous pregnancy (**Supplementary Table 10**). Thus, the findings of no patency did not exclude the chance of pregnancy.

## Discussion

### Main findings and interpretation

Our study confirms that OSCM HSG is followed by high pregnancy rates in women under 40 years. The proportion of women (40 years and below) who conceived within 6 months of OSCM HSG and successfully progressed to a live birth in our study was similar to that of the H2Oil trial, which used this age limit (36.6% vs 38.8%) (3). Similarly, the timing of pregnancy following the HSG was also consistent with previous studies (23, 24).

Interestingly, almost 30% of our cohort had iodine deficiency or insufficiency. The New Zealand soil is deficient in iodine (25), and fortification of bread with iodized salt is mandatory (26). However, mild iodine deficiency persists in the New Zealand population, especially women (27, 28), and iodine supplementation is recommended for women trying to conceive (29). In our study, we allowed the women to continue the iodine supplements (150 μg iodine/tablet) or multivitamin supplements (220 μg iodine/tablet) as advised by their respective fertility specialists. Our observations of baseline iodine status in this cohort suggest that despite the iodine fortification programmes in New Zealand (30) and the recommendations for iodine supplementation in women planning pregnancy (29), this issue remains an aspect of antenatal care that needs to be addressed. The pivotal role of iodine in conception and successful pregnancy progression, and the importance of achieving at least normal iodine levels has been demonstrated in other studies (16, 31, 32). It seems possible that iodine deficiency was one of the factors contributing to idiopathic infertility in this cohort of women. Thus, additional approaches to improve iodine status should be considered, including prescribing oral iodine supplements to women who are trying to conceive and adopting methods that can improve adherence, such as the use of one-dose or long-acting iodine replacements [e.g., oral OSCM (11)].

Not only were there persistent and high iodine levels following OSCM HSG, but those women with lower iodine levels at baseline were more likely to conceive following the procedure, indicating that treating iodine insufficiency/deficiency via the OSCM HSG’s iodine load improved fertility. Of note, the magnitude of iodine excess post-HSG did not correlate with pregnancy success, and we hypothesize that the correction of this iodine deficiency is more important than the extremely high levels subsequently achieved following the OSCM HSG. The reduced pregnancy rate in women with iodine excess at baseline was interesting and may reflect a cohort in whom other pathologies unaffected by iodine status are the cause of infertility. However, a better pregnancy rate in those who were iodine-sufficient (compared to the iodine-excess group) does raise the possibility that fertility can be enhanced with higher (supraphysiologic, yet not extreme) iodine levels in women with infertility. This question needs further exploration in future studies.

We also observed that women treated for mild SCH with levothyroxine during the six-month study period had higher pregnancy rates than those who were untreated. The treatment of mild SCH remains controversial (22). However, some studies suggest that SCH reduces fertility and that treatment improves pregnancy rates (33, 34). In this context, our findings suggest that women with mild SCH post-HSG who are attempting pregnancy may benefit from replacement therapy with levothyroxine.

This study demonstrates the limited fertility benefit of OSCM HSG in women aged 40 years or above, with only 16% conceiving and only one live birth recorded. This result is not surprising, reflecting the impact of aging and reduced follicular number. Indeed, age was the single most important factor in predicting pregnancy. The high miscarriage rates in this study should be interpreted in the context of an infertile cohort, which included women of older age, who had endometriosis, and/or experienced recurrent miscarriages. A previous large prospective Australian cohort study (5806 women, 31-36 years) had reported that the miscarriage rates varied highly between different groups of women, with a calculable rate of miscarriage ranging from 11.3 to 86.5 miscarriages per 100 live births (35). One study in younger women (18–33-year-olds) reported a lower miscarriage rate of 16% (36), whereas another earlier study including older women (aged 16–59 years) reported miscarriage rates of 33.4% (37). As expected, younger age was associated with higher AMH levels (38–40), a marker of follicular number, and predicted improved pregnancy rates following OSCM HSG. Thus, whilst OSCM HSG is a very good modality for augmenting fertility, the efficacy in those over 40 years appears limited.

### Limitations

Potentially important factors such as BMI and infertility aetiology could not be obtained for all participants, as these data were extracted retrospectively from clinical charts. BMI in particular, is known to adversely affect both female (41, 42) and male (43) fertility. While we had no data on the male partner’s BMI, among the 78% of study participants with BMI data, there was no evidence to suggest a BMI effect on fertility. Also, since 72% of study participants did not undergo any fertility treatment, it was not possible to carry out any robust analyses looking at the potential associations between assisted reproductive technologies and pregnancy rates. In addition, as most of our participants were recruited from private fertility clinics, disadvantaged groups were underrepresented, particularly women from Māori and Pacific communities. Thus, it is not possible to generalize our findings on iodine status to the entire female population of New Zealand or all women with infertility. Lastly, it is unknown if any of our study participants underwent transvaginal ultrasound or hysteroscopy before the OSCM HSG procedure to detect uterine or endometrial pathology. However, most study participants underwent transvaginal ultrasound following the OSCM HSG, and all of these were normal. Moreover, no uterine pathology was observed on fluoroscopy during the OSCM HSG.

### Strengths

To our knowledge, this is the only study that has examined the associations between HSG and fertility accounting for the women’s iodine levels before and after the procedure. This study highlights the caveats in iodine supplementation and the importance of ensuring prescription and compliance in women planning pregnancy. Our data suggest that iodine deficiency could contribute to some cases of unexplained infertility, and correction of iodine deficiency following OSCM exposure seems to be a contributing factor to improved fertility. Moreover, we show that fertility rates were markedly lower in women aged ≥40 years compared to younger women. These data provide additional evidence to fertility specialists and infertile couples for their decision-making process on whether to offer or undergo OSCM HSG, respectively.

### Conclusions

This study confirmed that while pregnancy rates were similar to other recent studies using OSCM HSG, women over 40 years of age have poor fertility outcomes. Iodine deficiency was relatively common despite government-instituted iodine fortification programmes and recommendations for iodine supplements by the fertility specialists. Interestingly, the fertility improvement with OSCM HSG was greater in those who were iodine deficient. We hypothesise that increased iodine levels may contribute to this procedure’s improved fertility. Treatment of the subclinical hypothyroidism that can occur following the OSCM HSG may also improve fertility rates further. Further studies are required to examine the potential effects of iodine deficiency on infertility, particularly the fertility improvement with OSCM HSG in iodine-deficient women. It would be interesting to determine if one oral or IM dose of OSCM is a suitable alternative to improve iodine levels and, subsequently, fertility. The benefit of OSCM HSG as a standalone fertility treatment and as an adjunct before intrauterine insemination or *in vitro* fertilization also needs to be explored further.

## Data availability statement

The study data cannot be made available in a public repository due to the strict conditions of the ethics approval, as no consent was obtained from study participants to make their confidential health data publicly available, even if anonymised. Nonetheless, the anonymised data on which this study was based could be made available to other investigators upon bona fide request, following all the necessary approvals (including ethics approval) of the detailed study proposal and statistical analysis plan. Any queries should be directed to Prof Paul Hofman (p.hofman@auckland.ac.nz).

## Ethics statement

Ethics approval for the SELFI (Safety and Efficacy of Lipiodol in Fertility Investigations) Study was granted by the Northern B Health and Disability Ethics Committee (19/NTB/52). The studies were conducted in accordance with the local legislation and institutional requirements. The participants provided their written informed consent to participate in this study.

## Author contributions

DM: Data curation, Formal analysis, Investigation, Methodology, Resources, Writing – original draft, Writing – review & editing. JP: Conceptualization, Resources, Supervision, Writing – review & editing. RS: Conceptualization, Resources, Supervision, Writing – review & editing. NJ: Conceptualization, Resources, Supervision, Writing – review & editing. SO’S: Conceptualization, Resources, Supervision, Writing – review & editing. JD: Data curation, Formal analysis, Writing – original draft, Writing – review & editing. PH: Conceptualization, Formal analysis, Funding acquisition, Methodology, Resources, Supervision, Writing – review & editing.

## References

[1] Alper MM Garner PR Spence JE Quarrington AM. Pregnancy rates after hysterosalpingography with oil- and water-soluble contrast media. Obstet Gynecol. (1986) 68:6–9. 3014408

[2] de Boer AD Vemer HM Willemsen WN Sanders FB. Oil or aqueous contrast media for hysterosalpingography: a prospective, randomized, clinical study. Eur J Obstet Gynecol Reprod Biol. (1988) 28:65–8. doi: 10.1016/0028-2243(88)90060-3 2839382

[3] Dreyer K van Rijswijk J Mijatovic V Goddijn M Verhoeve HR van Rooij IAJ. Oil-based or water-based contrast for hysterosalpingography in infertile women. N Engl J Med. (2017) 376:2043–52. doi: 10.1056/NEJMoa1612337 28520519

[4] Johnson NP Kwok R Stewart AW Saththianathan M Hadden WE Chamley LW. Lipiodol fertility enhancement: two-year follow-up of a randomized trial suggests a transient benefit in endometriosis, but a sustained benefit in unexplained infertility. Hum Reprod. (2007) 22:2857–62. doi: 10.1093/humrep/dem275 17890725

[5] Rasmussen F Justesen P Tonner Nielsen D. Therapeutic value of hysterosalpingography with Lipiodol Ultra Fluid. Acta Radiol. (1987) 28:319–22. doi: 10.1177/028418518702800318 2820454

[6] van Rijswijk J van Welie N Dreyer K Tajik P Lambalk CB Hompes P. Tubal flushing with oil- or water-based contrast medium: can we identify markers that indicate treatment benefit? Hum Reprod Open. (2019) 2019:hoz015. doi: 10.1093/hropen/hoz015 31334364 PMC6638263

[7] van Welie N Dreyer K van Rijswijk J Verhoeve HR Goddijn M Nap AW. Treatment effect of oil-based contrast is related to experienced pain at HSG: a *post-hoc* analysis of the randomised H2Oil study. Hum Reprod. (2019) 34:2391–8. doi: 10.1093/humrep/dez206 PMC6995245 31887222

[8] Izumi G Koga K Takamura M Bo W Nagai M Miyashita M. Oil-soluble contrast medium (OSCM) for hysterosalpingography modulates dendritic cell and regulatory T cell profiles in the peritoneal cavity: A possible mechanism by which OSCM enhances fertility. J Immunol. (2017) 198:4277–84. doi: 10.4049/jimmunol.1600498 28455434

[9] Johnson N Baidya S Jessup S Print C Muthukaruppan A Chamley L. Randomised trial of Lipiodol Uterine Bathing Effect (LUBE) in women with endometriosis-related infertility. Fertil Reprod. (2019) 1:57–64. doi: 10.1142/S2661318219500063

[10] Mathews DM Johnson NP Sim RG O'Sullivan S Peart JM Hofman PL. Iodine and fertility: do we know enough? Hum Reprod. (2021) 36:265–74. doi: 10.1093/humrep/deaa312 33289034

[11] Leverge R Bergmann JF Simoneau G Tillet Y Bonnemain B. Bioavailability of oral vs intramuscular iodinated oil (Lipiodol UF) in healthy subjects. J Endocrinol Invest. (2003) 26:20–6. 12762636

[12] Miyamoto Y Tsujimoto T Iwai K Ishida K Uchimoto R Miyazawa T. Safety and pharmacokinetics of iotrolan in hysterosalpingography. Retention and irritability compared with Lipiodol. Invest Radiol. (1995) 30:538–43. doi: 10.1097/00004424-199509000-00005 8537211

[13] Mathews DM Peart JM Sim RG Johnson NP O'Sullivan S Derraik JGB. The SELFI Study: Iodine excess and thyroid dysfunction in women undergoing oil-soluble contrast hysterosalpingography. J Clin Endocrinol Metab. (2022) 107:3252–60. doi: 10.1210/clinem/dgac546 PMC9693785 36124847

[14] Slebodziński AB. Ovarian iodide uptake and triiodothyronine generation in follicular fluid. The enigma of the thyroid ovary interaction. Domest Anim Endocrinol. (2005) 29:97–103. doi: 10.1016/j.domaniend.2005.02.029 15927769

[15] Riesco-Eizaguirre G Leoni SG Mendiola M Estevez-Cebrero MA Gallego MI Redondo A. NIS mediates iodide uptake in the female reproductive tract and is a poor prognostic factor in ovarian cancer. J Clin Endocrinol Metab. (2014) 99:E1199–208. doi: 10.1210/jc.2013-4249 24708099

[16] Mills JL Buck Louis GM Kannan K Weck J Wan Y Maisog J. Delayed conception in women with low-urinary iodine concentrations: a population-based prospective cohort study. Hum Reprod. (2018) 33:426–33. doi: 10.1093/humrep/dex379 PMC6454505 29340704

[17] Mathews DM Peart JM Sim RG Johnson NP O'Sullivan S Derraik JGB. The effect of acute and chronic iodine excess on thyroid profile and reproductive function of women using Lipiodol during hysterosalpingography and the potential impact on thyroid function of their offspring: The SELFI study protocol. Med Case Rep Study Protoc. (2021) 2:e0148. doi: 10.1097/md9.0000000000000148

[18] Zou G. A modified poisson regression approach to prospective studies with binary data. Am J Epidemiol. (2004) 159:702–6. doi: 10.1093/aje/kwh090 15033648

[19] Rothman KJ. No adjustments are needed for multiple comparisons. Epidemiology. (1990) 1:43–6. doi: 10.1097/00001648-199001000-00010 2081237

[20] Barnhart KT. Live birth is the correct outcome for clinical trials evaluating therapy for the infertile couple. Fertil Steril. (2014) 101:1205-8. doi: 10.1016/j.fertnstert.2014.03.026 PMC4040520 24786740

[21] WHO/UNICEF/ICCIDD. Assessment of iodine deficiency disorders and monitoring their elimination: a guide for programme managers. 3rd ed. Geneva: World Health Organization (2007).

[22] Javed Z Sathyapalan T. Levothyroxine treatment of mild subclinical hypothyroidism: a review of potential risks and benefits. Ther Adv Endocrinol Metab. (2016) 7:12–23. doi: 10.1177/2042018815616543 26885359 PMC4740939

[23] Reindollar RH Regan MM Neumann PJ Levine BS Thornton KL Alper MM. A randomized clinical trial to evaluate optimal treatment for unexplained infertility: the fast track and standard treatment (FASTT) trial. Fertil Steril. (2010) 94:888–99. doi: 10.1016/j.fertnstert.2009.04.022 19531445

[24] Johnson NP. Review of lipiodol treatment for infertility - an innovative treatment for endometriosis-related infertility? Aust N Z J Obstet Gynaecol. (2014) 54:9–12. doi: 10.1111/ajo.12141 24138402

[25] Hercus CE Benson WN Carter CL. Endemic goitre in New Zealand, and its relation to the soil-iodine: Studies from the University of Otago, New Zealand. J Hyg. (1925) 24:321–402.3. doi: 10.1017/s0022172400008779 20474868 PMC2167656

[26] Pettigrew-Porter A Skeaff S Gray A Thomson C Croxson M. Are pregnant women in New Zealand iodine deficient? A cross-sectional survey. Aust N Z J Obstet Gynaecol. (2011) 51:464–7. doi: 10.1111/ajo.2011.51.issue-5 21875425

[27] Ministry of Health. Biomedical Data Explorer 2014/15: New Zealand Health Survey (2020). Available online at: https://minhealthnz.shinyapps.io/nz-health-survey-2014-15-biomedical.

[28] Brough L Jin Y Shukri NH Wharemate ZR Weber JL Coad J. Iodine intake and status during pregnancy and lactation before and after government initiatives to improve iodine status, in Palmerston North, New Zealand: a pilot study. Matern Child Nutr. (2015) 11:646–55. doi: 10.1111/mcn.12055 PMC6860324 23782592

[29] Alexander EK Pearce EN Brent GA Brown RS Chen H Dosiou C. 2017 Guidelines of the American Thyroid Association for the diagnosis and management of thyroid disease during pregnancy and the postpartum. Thyroid. (2017) 27:315–89. doi: 10.1089/thy.2016.0457 28056690

[30] Edmonds JC McLean RM Williams SM Skeaff SA. Urinary iodine concentration of New Zealand adults improves with mandatory fortification of bread with iodised salt but not to predicted levels. Eur J Nutr. (2016) 55:1201–12. doi: 10.1007/s00394-015-0933-y 26018655

[31] Mills JL Ali M Buck Louis GM Kannan K Weck J Wan Y. Pregnancy loss and iodine status: The LIFE prospective cohort study. Nutrients. (2019) 11:534. doi: 10.3390/nu11030534 30823683 PMC6471412

[32] Dillon JC Milliez J. Reproductive failure in women living in iodine deficient areas of West Africa. BJOG. (2000) 107:631–6. doi: 10.1111/j.1471-0528.2000.tb13305.x 10826578

[33] Yoshioka W Amino N Ide A Kang S Kudo T Nishihara E. Thyroxine treatment may be useful for subclinical hypothyroidism in patients with female infertility. Endocr J. (2015) 62:87–92. doi: 10.1507/endocrj.EJ14-0300 25312747

[34] Verma I Sood R Juneja S Kaur S. Prevalence of hypothyroidism in infertile women and evaluation of response of treatment for hypothyroidism on infertility. Int J Appl Basic Med Res. (2012) 2:17–9. doi: 10.4103/2229-516x.96795 PMC3657979 23776802

[35] Hure AJ Powers JR Mishra GD Herbert DL Byles JE Loxton D. Miscarriage, preterm delivery, and stillbirth: large variations in rates within a cohort of Australian women. PLoS One. (2012) 7:e37109. doi: 10.1371/journal.pone.0037109 22629355 PMC3357437

[36] Herbert D Lucke J Dobson A. Pregnancy losses in young Australian women: findings from the Australian Longitudinal Study on Women's Health. Womens Health Issues. (2009) 19:21–9. doi: 10.1016/j.whi.2008.08.007 19111784

[37] Smith AM Rissel CE Richters J Grulich AE de Visser RO. Sex in Australia: reproductive experiences and reproductive health among a representative sample of women. Aust N Z J Public Health. (2003) 27:204–9. doi: 10.1111/j.1467-842X.2003.tb00809.x 14696712

[38] van Rooij IA Broekmans FJ te Velde ER Fauser BC Bancsi LF de Jong FH. Serum anti-Müllerian hormone levels: a novel measure of ovarian reserve. Hum Reprod. (2002) 17:3065–71. doi: 10.1093/humrep/17.12.3065 12456604

[39] Meczekalski B Czyzyk A Kunicki M Podfigurna-Stopa A Plociennik L Jakiel G. Fertility in women of late reproductive age: the role of serum anti-Müllerian hormone (AMH) levels in its assessment. J Endocrinol Invest. (2016) 39:1259–65. doi: 10.1007/s40618-016-0497-6 PMC5069312 27300031

[40] Dewailly D Andersen CY Balen A Broekmans F Dilaver N Fanchin R. The physiology and clinical utility of anti-Mullerian hormone in women. Hum Reprod Update. (2014) 20:370–85. doi: 10.1093/humupd/dmt062 24430863

[41] Pandey S Pandey S Maheshwari A Bhattacharya S. The impact of female obesity on the outcome of fertility treatment. J Hum Reprod Sci. (2010) 3:62–7. doi: 10.4103/0974-1208.69332 PMC2970793 21209748

[42] Rittenberg V Seshadri S Sunkara SK Sobaleva S Oteng-Ntim E El-Toukhy T. Effect of body mass index on IVF treatment outcome: an updated systematic review and meta-analysis. Reprod BioMed Online. (2011) 23:421–39. doi: 10.1016/j.rbmo.2011.06.018 21885344

[43] Mushtaq R Pundir J Achilli C Naji O Khalaf Y El-Toukhy T. Effect of male body mass index on assisted reproduction treatment outcome: an updated systematic review and meta-analysis. Reprod BioMed Online. (2018) 36:459–71. doi: 10.1016/j.rbmo.2018.01.002 29452915

