# Supplementary material for: Iodine and other factors associated with fertility outcome following oil-soluble contrast medium hysterosalpingography: a prospective cohort study
Source: Front Endocrinol (Lausanne). 2024 Jun 20;15:1257888. doi: 10.3389/fendo.2024.1257888 (PMC11224778; doi:10.3389/fendo.2024.1257888)
Supplement: Supplementary file 1 [file DataSheet_1.pdf]

Supplementary File

Iodine and other factors associated with fertility outcome following oil-soluble contrast medium hysterosalpingography: A prospective cohort study

Divya M Mathews[1,2], Jane M Peart[3], Robert G Sim[3], Neil P Johnson[4,5,6], Susannah O'Sullivan[7], José G B Derraik[8,9,10], Paul L Hofman[1,2*]

[1] Liggins Institute, University of Auckland, Auckland, New Zealand

[2] Starship Children's Hospital, Health New Zealand | Te Whatu Ora Auckland, New Zealand

[3] Auckland Radiology Group, Auckland, New Zealand

[4] Robinson Research Institute, University of Adelaide, Adelaide, Australia

[5] Department of Obstetrics and Gynaecology, Faculty of Medical and Health Sciences, University of Auckland, Auckland, New Zealand

[6] Repromed Auckland, Auckland, New Zealand

[7] Endocrinology, Greenlane Clinical Centre, Auckland District Health Board, Auckland, New Zealand

[8] Department of Paediatrics: Child & Youth Health, Faculty of Medicine and Health Sciences, University of Auckland, Auckland, New Zealand

[9] Environmental–Occupational Health Sciences and Non-Communicable Diseases Research Group, Research Institute for Health Sciences, Chiang Mai University, Chiang Mai, Thailand

[10] Department of Women's and Children's Health, Uppsala University, Uppsala, Sweden

* Corresponding author: Liggins Institute, University of Auckland, Private Bag 92019, Auckland 1142, New Zealand;

Email: p.hofman@auckland.ac.nz

Supplementary Table 1

Incidence of thyroid dysfunction at baseline and within six months after undergoing oil-soluble contrast medium (OSCM) hysterosalpingography (HSG).

THYROID DYSFUNCTION		BASELINE	AFTER HSG [A]
**Subclinical hypothyroidism**	**TSH >4 mIU/L (all)**	8/196 (4.1%)	71/188 (37.8%)[B]
	**TSH >4 to ≤10 mIU/L**	8/8	68/71
	**TSH >10 mIU/L**	nil	3/71
**Overt hypothyroidism**		n/a	nil
**Subclinical hyperthyroidism**		n/a	4/196 (2.0%)
**Overt hyperthyroidism**		n/a	5/196 (2.6%)

Table originally published in Mathews et al. The SELFI Study: Iodine excess and thyroid dysfunction in women undergoing oil-soluble contrast hysterosalpingography. J Clin Endocrinol Metab 2022; 107: 3252-60.

Abbreviations: FT4, free thyroxine; HSG, hysterosalpingography; n/a, not applicable as subclinical and overt hypothyroidism were exclusion criteria for this study; TSH, thyrotropin.

Subclinical hypothyroidism: TSH >4 mIU/L and FT4 ≥11 to ≤22 pmol/L at any assessment; Overt hypothyroidism: TSH >4 mIU/L and FT4 <11 pmol/L at any assessment post-HSG; Subclinical hyperthyroidism: TSH<0.3 mIU/L and FT4 ≥11 to ≤22 pmol/L at any assessment; Overt hyperthyroidism: TSH <0.3 and FT4 >22 pmol/L at any assessment.

[A] Excluding the visits at or after a participant became pregnant, and visits at or after participants started on thyroxine treatment, where relevant.

[B] Excluding those listed in footnote a above as well as participants with baseline subclinical hypothyroidism.

Supplementary Table 2

Inclusion and exclusion criteria for the SELFI Study.

**Inclusion**	**1.** Being of reproductive age (i.e., 15 to <50 years); AND
	**2.** Having primary or secondary infertility; AND
	**3.** Referred for OSCM HSG in the Auckland region; AND
	**4a.** Have probable tubal patency, based on the criterion of "low risk for pre-existing tubal damage" as per Dreyer et al.[1]; OR<br>**4b.** If known to have damage to one or both fallopian tubes, having a referral for an OSCM HSG advised by the fertility specialist (for fertility enhancement from uterine bathing effect of Lipiodol[2]); AND
	**5a.** Have normal thyroid function (i.e., normal non-pregnant free T4 and TSH); OR<br>**5b.** Have subclinical hypothyroidism (defined as a TSH > 4mIU/L and normal free T4 levels), and not be on treatment with levothyroxine.
**Exclusion**	**1.** Have any known contraindication for OSCM use (poppy seed or poppy seed oil allergy, or history of reactions to iodine); OR
	**2.** Have active thyroid disease, such as overt hypothyroidism, hyperthyroidism, or thyroid cancer; OR
	**3.** Currently receive treatment with levothyroxine or antithyroid medications; OR
	**4.** On medications known to affect thyroid function or iodine metabolism (e.g., lithium, amiodarone, etc.); OR
	**5.** Have used water-soluble contrast within three months prior to the OSCM HSG procedure; OR
	**6.** Have used OSCM or any other oil-soluble contrast medium within six months before the procedure.

HSG, hysterosalpingography; OSCM, oil-soluble contrast medium; SELFI, Safety and Efficacy of Lipiodol in Fertility Investigations; T4, tetraiodothyronine or thyroxine; TSH, thyroid stimulating hormone.

Adapted from: Mathews et al. The effect of acute and chronic iodine excess on thyroid profile and reproductive function of women using Lipiodol during hysterosalpingography and the potential impact on thyroid function of their offspring: The SELFI study protocol. Med Case Rep Study Protoc 2021; 2: e0148.

[1] Dreyer K et al. Oil-based or water-based contrast for hysterosalpingography in infertile women. N Eng J Med 2017;376:2043–52.

[2] Reilly et al. The IVF-LUBE trial - a randomized trial to assess Lipiodol® uterine bathing effect in women with endometriosis or repeat implantation failure undergoing IVF. Reprod Biomed Online 2019;38:380–6.

Supplementary Table 3

Biochemical pregnancy rates by infertility cause based on beta human chorionic gonadotropin (β-hCG) positivity.

BIOCHEMICAL PREGNANCY	INFERTILITY CAUSE		
	Endometriosis	Idiopathic/Other	PCOS
n	37	144	15
**β-hCG negative**	20 (54%)	83 (58%)	10 (67%)
**β-hCG positive**	17 (46%)	61 (42%)	5 (33%)

Data are *n* (%). PCOS, polycystic ovarian syndrome.
p=0.73 for the comparison of β-hCG positivity rates across infertility groups from a Fisher's exact test.

Mathews DM, Jane M Peart JM, Sim RG, Johnson NP, O'Sullivan S, Derraik JGB, Hofman PL. Iodine and other factors associated with fertility outcome following oil-soluble contrast medium hysterosalpingography: A prospective cohort study. *Frontiers in Endocrinology* 2024; 15: 1257888. doi: 10.3389/fendo.2024.1257888

Supplementary Table 4

Recorded urine iodine status at baseline according to the woman's cause of infertility.

UIC STATUS AT ENTRY	INFERTILITY CAUSE		
	Endometriosis	Idiopathic/Other	PCOS
n	37	144	15
**Deficient**	8 (25%)	39 (29%)	6 (40%)
**Normal**	18 (56%)	77 (57%)	6 (40%)
**Excessive**	6 (19%)	20 (15%)	3 (20%)

Data are *n* (%). PCOS, polycystic ovarian syndrome; UIC, urine iodine concentration.
Iodine status was defined based on UIC as per WHO criteria: deficient (<100 μg/L), sufficient (≥100 to <300 μg/L), and excessive (≥300 μg/L) (WHO/UNICEF/ICCIDD. Assessment of Iodine Deficiency Disorders and Monitoring Their Elimination: A Guide for Programme Managers, 3rd ed. World Health Organization; 2007).
p=0.67 for the comparison of UIC status at baseline between infertility groups from a Fisher's exact test.

Supplementary Table 5

Biochemical pregnancy rates by infertility cause and baseline urine iodine status.

UIC STATUS AT ENTRY	BIOCHEMICAL PREGNANCY	INFERTILITY CAUSE		
		**Endometriosis**	**Idiopathic/Other**	**PCOS**
**Deficient**	***n***	**8**	**39**	**6**
	**β-hCG negative**	4 (50%)	20 (51%)	4 (67%)
	**β-hCG positive**	4 (50%)	19 (49%)	2 (33%)
**Normal**	***n***	**18**	**77**	**6**
	**β-hCG negative**	10 (56%)	44 (57%)	4 (67%)
	**β-hCG positive**	8 (44%)	33 (43%)	2 (33%)
**Excessive**	***n***	**6**	**20**	**3**
	**β-hCG negative**	3 (50%)	15 (75%)	2 (67%)
	**β-hCG positive**	3 (50%)	5 (25%)	1 (33%)
***p*-value ***		>0.99	0.22	>0.99

Data are *n* (%). PCOS, polycystic ovarian syndrome; UIC, urine iodine concentration.
Biochemical pregnancy was based on beta human chorionic gonadotropin (β-hCG) positivity. Iodine status was defined based on UIC as per WHO criteria: deficient (<100 µg/L), sufficient (≥100 to <300 µg/L), and excessive (≥300 µg/L) (WHO/UNICEF/ICCIDD. Assessment of Iodine Deficiency Disorders and Monitoring Their Elimination: A Guide for Programme Managers, 3rd ed. World Health Organization; 2007).
**p*-values for the comparison of β-hCG positivity rates according to UIC status at baseline within a given infertility group derived from Fisher's exact tests.

Mathews DM, Jane M Peart JM, Sim RG, Johnson NP, O'Sullivan S, Derraik JGB, Hofman PL. Iodine and other factors associated with fertility outcome following oil-soluble contrast medium hysterosalpingography: A prospective cohort study. *Frontiers in Endocrinology* 2024; 15: 1257888. doi: 10.3389/fendo.2024.1257888

Supplementary Table 6

Body mass index (BMI) in women who attained or did not attain biochemical pregnancy.

		Biochemical pregnancy		
**Parameter**	**Levels**	**β-hCG negative**	**β-hCG positive**	***p*-value**
**Total *n***		113	83	–
***n* missing (no BMI data)**		27 (24%)*	17 (20%)*	–
***n* with BMI data**		86 (76%)*	66 (80%)*	–
**BMI at baseline (kg/m^2)**		25.1 ± 4.0	24.0 ± 3.7	0.10
**BMI status**	**Normal weight**	50 (52%)†	46 (48%)†	0.18
	**Overweight/obesity**	36 (64%)†	20 (36%)†	

Data are *n* (%) or mean ± standard deviation, as appropriate.
Biochemical pregnancy was based on beta human chorionic gonadotropin (β-hCG) positivity.
Normal weight, BMI ≥18.5 to <25 kg/m^2; overweight/obesity, BMI ≥25 kg/m^2.
P-values were derived from a one-way ANOVA (BMI) or a Fisher's exact test (BMI status).
* Percentages within a given β-hCG result (i.e., column).
† Percentages within a given BMI status (i.e., row).

Mathews DM, Jane M Peart JM, Sim RG, Johnson NP, O'Sullivan S, Derraik JGB, Hofman PL. Iodine and other factors associated with fertility outcome following oil-soluble contrast medium hysterosalpingography: A prospective cohort study. *Frontiers in Endocrinology* 2024; 15: 1257888. doi: 10.3389/fendo.2024.1257888

Supplementary Table 7

Biochemical pregnancy rates by fertility treatment based on beta human chorionic gonadotropin (β-hCG) positivity.

BIOCHEMICAL PREGNANCY	FERTILITY TREATMENT		
	None	IUI	IVF
n	141	19	31
**β-hCG negative**	79 (56%)	10 (53%)	30 (65%)
**β-hCG positive**	62 (44%)	9 (47%)	11 (35%)

Data are *n* (%); five study participants with missing data on fertility treatment were excluded.
IUI, intrauterine insemination; IVF, in vitro fertilisation.
p=0.65 for the comparison of β-hCG positivity rates across fertility treatments from a Fisher's exact test.

Mathews DM, Jane M Peart JM, Sim RG, Johnson NP, O'Sullivan S, Derraik JGB, Hofman PL. Iodine and other factors associated with fertility outcome following oil-soluble contrast medium hysterosalpingography: A prospective cohort study. *Frontiers in Endocrinology* 2024; 15: 1257888. doi: 10.3389/fendo.2024.1257888

Supplementary Table 8

Biochemical pregnancy and ongoing pregnancy rates by women's age at baseline.

	Age at baseline		
	**<35 years**	**35–39.9 years**	**≥40 years**
***n* (%)**	79 (40%)	79 (40%)	38 (19%)
**β-hCG negative**	39 (49%)	42 (53%)	32 (84%)
**β-hCG positive**	40 (51%)	37 (47%)	6 (16%)
**Ongoing pregnancy ***	34 (85%)	24 (65%)	1 (17%)
**Miscarriage ***	6 (15%)	13 (35%)	5 (83%)

Data are *n* (%). Biochemical pregnancy was based on beta human chorionic gonadotropin (β-hCG) positivity.
$P<0.001$ for the difference in β-hCG positivity rates from a Fisher's exact test.
*Proportions out of the number of women who became pregnant during the study.

Mathews DM, Jane M Peart JM, Sim RG, Johnson NP, O'Sullivan S, Derraik JGB, Hofman PL. Iodine and other factors associated with fertility outcome following oil-soluble contrast medium hysterosalpingography: A prospective cohort study. *Frontiers in Endocrinology* 2024; 15: 1257888. doi: 10.3389/fendo.2024.1257888

Supplementary Table 9

Iodine status and related baseline parameters in women who became pregnant or not after undergoing oil-soluble contrast medium (OSCM) hysterosalpingography (HSG).

PARAMETER	NO PREGNANCY	PREGNANCY	*P*-VALUE
**n (%) ***	106 (58%)	77 (42%)	
**Age (years)**	36.9 [33.8, 40.3]	35.1 [32.0, 37.6]	**0.003**
**Urine iodine (μg/L)**	158 (134, 183)	123 (105, 145)	**0.042**
**TSH (mIU/L)**	1.8 [1.3, 2.3]	1.8 [1.3, 2.7]	0.15

TSH, thyroid-stimulating hormone. Pregnancy was determined based on beta human chorionic gonadotropin positivity.

* Percentages from the overall study population with iodine data at baseline (n=183).

Age and TSH data are medians [quartile 1, quartile 3], while data on urine iodine were log-transformed for analyses and are reported here as the back-transformed means and 95% confidence intervals. *p*-values for between-group comparisons statistically significant at $p<0.05$ are shown in bold.

Mathews DM, Jane M Peart JM, Sim RG, Johnson NP, O'Sullivan S, Derraik JGB, Hofman PL. Iodine and other factors associated with fertility outcome following oil-soluble contrast medium hysterosalpingography: A prospective cohort study. *Frontiers in Endocrinology* 2024; 15: 1257888. doi: 10.3389/fendo.2024.1257888

Supplementary Table 10

Biochemical pregnancy rates by tubal patency status based on beta human chorionic gonadotropin (β-hCG) positivity.

	Tubal patency status		
**Biochemical pregnancy**	**Bilateral**	**Unilateral**	**None**
n	136	41	16
**β-hCG negative**	69 (51%)	30 (73%)	13 (81%)
**β-hCG positive**	67 (49%)	11 (27%)	3 (19%)

Data are *n* (%); three study participants with unconfirmed data on tubal patency were excluded.
P=0.006 for the comparison of β-hCG positivity rates according to tubal patency status from a Fisher's exact test.

Mathews DM, Jane M Peart JM, Sim RG, Johnson NP, O'Sullivan S, Derraik JGB, Hofman PL. Iodine and other factors associated with fertility outcome following oil-soluble contrast medium hysterosalpingography: A prospective cohort study. *Frontiers in Endocrinology* 2024; 15: 1257888. doi: 10.3389/fendo.2024.1257888

Supplementary Figure 1

Diagram showing the flow of participants throughout the SELFI Study and the timing of the various clinical investigations.

Abbreviations: FT3, free triiodothyronine; FT4, free tetraiodothyronine; HSG, hysterosalpingography; OSCM, oil-soluble contrast medium; SELFI, Safety and Efficacy of Lipiodol in Fertility Investigations; TSH, thyroid stimulating hormone; UIC, urine iodine concentration.
Reproduced from Mathews et al. The SELFI Study: Iodine excess and thyroid dysfunction in women undergoing oil-soluble contrast hysterosalpingography. J Clin Endocrinol Metab 2022; 107: 3252-60.

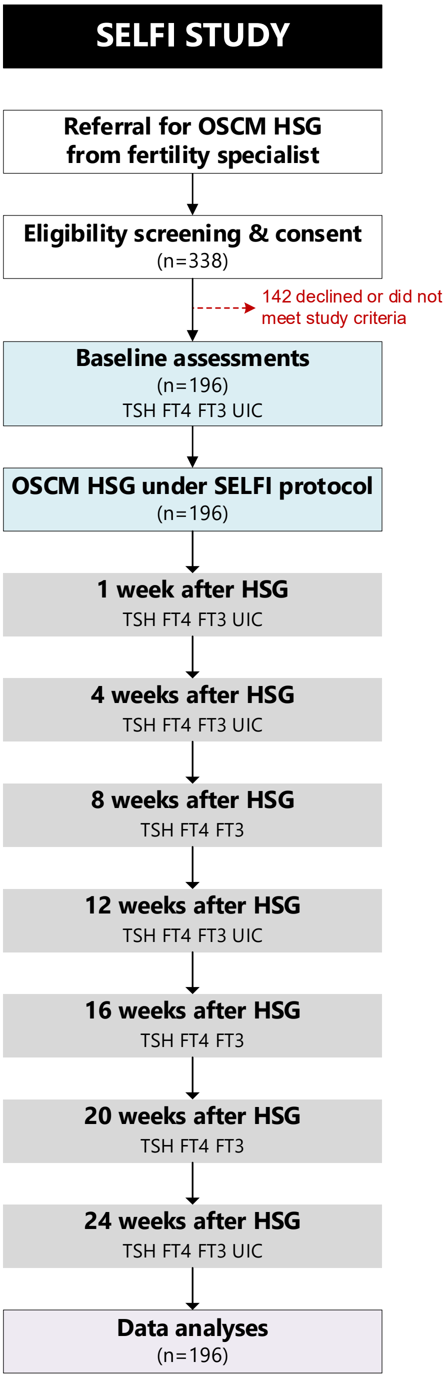

Mathews DM, Jane M Peart JM, Sim RG, Johnson NP, O'Sullivan S, Derraik JGB, Hofman PL. Iodine and other factors associated with fertility outcome following oil-soluble contrast medium hysterosalpingography: A prospective cohort study. *Frontiers in Endocrinology* 2024; 15: 1257888. doi: 10.3389/fendo.2024.1257888

Supplementary Figure 2

Linear association between anti-Müllerian hormone (AMH) concentrations at baseline and the women's age (n=188).

The diagonal black line is the fitted linear regression with its coefficient (r^2) and respective *p*-value also shown.

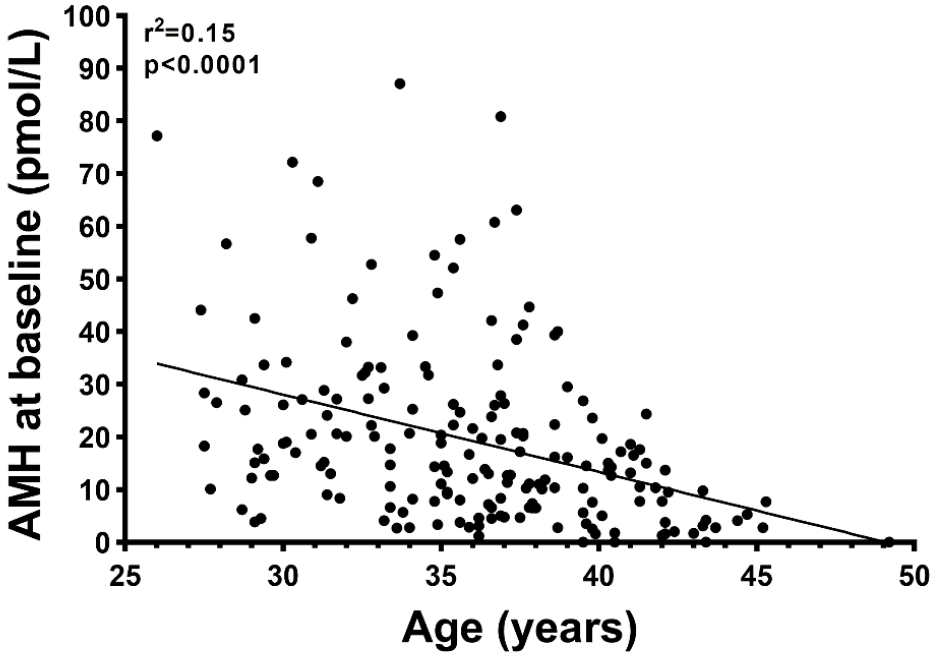

Mathews DM, Jane M Peart JM, Sim RG, Johnson NP, O'Sullivan S, Derraik JGB, Hofman PL. Iodine and other factors associated with fertility outcome following oil-soluble contrast medium hysterosalpingography: A prospective cohort study. *Frontiers in Endocrinology* 2024; 15: 1257888. doi: 10.3389/fendo.2024.1257888
